# Thrombospondin-1 modified bone marrow mesenchymal stem cells (BMSCs) promote neurite outgrowth and functional recovery in rats with spinal cord injury

**DOI:** 10.18632/oncotarget.22018

**Published:** 2017-10-24

**Authors:** Yujie Pu, Ke Meng, Chuanlong Gu, Linlin Wang, Xiaoming Zhang

**Affiliations:** ^1^ Department of Basic Medicine Sciences, School of Medicine, Zhejiang University, Hangzhou 310058, China

**Keywords:** spinal cord injury, bone marrow mesenchymal stem cells (BMSCs), thrombospondin-1 (TSP-1), neurite outgrowth, functional recovery

## Abstract

Stem cell therapies are currently gaining momentum in the treatment of spinal cord injury (SCI). However, unsatisfied intrinsic neurite growth capacity constitutes significant obstacles for injured spinal cord repair and ultimately results in neurological dysfunction. The present study assessed the efficacy of thrombospondin-1 (TSP-1), a neurite outgrowth-promoting molecule, modified bone marrow mesenchymal stem cells (BMSCs) on promoting neurite outgrowth *in vitro* and *in vivo* of Oxygen–Glucose Deprivation (OGD) treated motor neurons and SCI rat models. The present results demonstrated that the treatment of BMSCs+TSP-1 could promote the neurite length, neuronal survival, and functional recovery after SCI. Additionally, TSP-1 could activate transforming growth factor-β1 (TGF-β1) then induced the smad2 phosphorylation, and expedited the expression of GAP-43 to promote neurite outgrowth. The present study for the first time demonstrated that BMSCs+TSP-1 could promote neurite outgrowth and functional recovery after SCI partly through the TGF-β1/p-Samd2 pathway. The study provided a novel encouraging evidence for the potential treatment of BMSCs modification with TSP-1 in patients with SCI.

## INTRODUCTION

Spinal cord injury (SCI) becomes a major public health issue that impairs motor, sensory, autonomic function, and results in neurological dysfunctions in patients with SCI [1, 2]. A total of 233 patients were diagnosed with traumatic spinal cord injury during the study period or 26 per million annually on average in Iceland. Males were 73% and the mean age was 39 years old [3]. The pathology of SCI can be classified into primary and secondary injury. Primary injury often results from mechanical impaction to the spine. Secondary injury refers to the multifaceted pathological mechanisms that start after primary SCI and include a breakdown of blood-spinal cord barrier (BSCB), neuroinflammation, oxidative stress and neuronal apoptosis [4–6]. The pathology of secondary injury resulting from neural tissue destruction, poor microenvironment, and unsatisfied intrinsic neurite growth capacity can be at least as responsible for long-term dysfunction [7].

Thrombospondin-1 (TSP-1) is secreted by astrocytes in the central nervous system during development and inflammation [8, 9]. The scientists recently found that TSP-1 could increase the speed of synapse formation in cultured hippocampal neurons [10]. Moreover, TSPs are up regulated in reactive astrocytes following injury or disease, where they are well placed to modulate the repair processes such as tissue remodeling, neurite outgrowth, glial scar formation, and rewiring of neural circuitry [11, 12]. Thus, we proposed the hypothesis that increasing expression of TSP-1 in the injured spinal cord could promote neurite outgrowth and functional recovery after SCI.

Bone marrow mesenchymal stem cells (BMSCs) show interesting features, such as easy to harvest, culture, expand *in vitro*, immunomodulation, and neurotrophic activities [13–15]. Furthermore, the BMSCs transplantation has been tested to be helpful in neurological, cardiovascular, immunological disease [16]. The BMSCs can act as cellular vehicles for ideal gene delivery in gene therapy for some diseases [17–19]. Our previous studies established BMSCs isolation, culture and transplantation in the injured nervous tissues after SCI. We found that BMSCs could ameliorate neurological function after transplantation for SCI [20–22]. Other previous results revealed that BMSCs could support the cell survival through their secretion of neurotrophic macromolecules and improve the microenvironments, to protect injured neurons and functional recovery [20, 23, 24]. Although there were a large number of studies of cell-based therapy in the treatment of SCI, the poor outcomes were attributed mainly to suboptimal neurite regeneration [25–27]. Therefore, in the present study, we have performed for the first time to modify the BMSCs with the TSP-1, observe the effects of TSP-1 overexpression BMSCs on neurite outgrowth and functional improvement after SCI, and explore the possible mechanism involved in. The present study will lay a framework for the future exploration of promoting axon regeneration after SCI, and highlight a potential strategy for the treatment of TSP-1 on patients with SCI.

## RESULTS

### The BMSCs isolation and TSP-1 transfection

**Figure 1:**
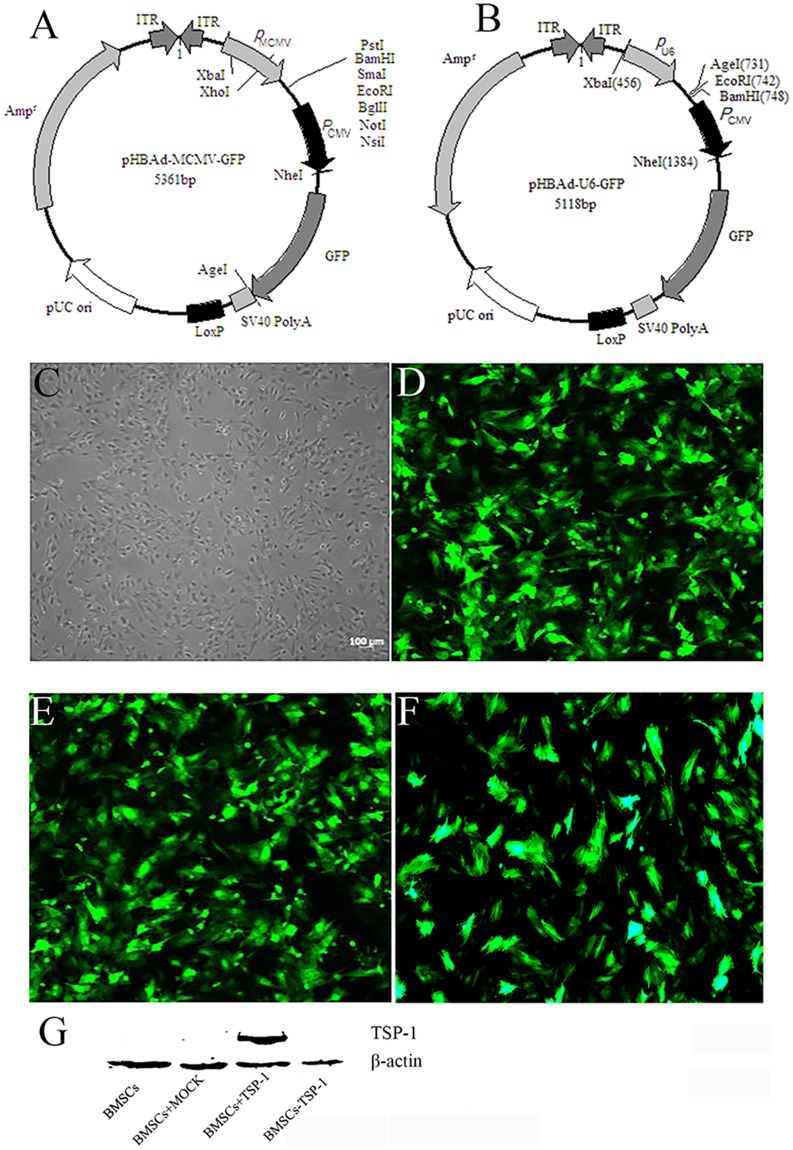
TheTSP-1 and TSP-1 siRNA plasmid construction and expression **(A)** Maps of pHBAd-MCMV-GFP. **(B)** Maps of pHBAd-U6-GFP. **(C)** Morphology characterization of cultured BMSCs (Light microscopy). **(D)** GFP expression in BMSCs 48 h after transduction of empty vector into BMSCs. **(E)** GFP expression in BMSCs 48 h after transduction of Adv-TSP-1 into BMSCs. **(F)** GFP expression in BMSCs 48 h after transduction of Adv-TSP-1-shRNA into BMSCs. Scale bar:100μm. **(G)** Western blotting detection of TSP-1 protein expression.

The primary BMSCs were isolated and identified as previously reporting [20, 21]. The pHBAd-MCMV-GFP over expression vector had double-promoter to assess the transfection efficacy and expression of TSP-1. The double promoter plasmids contained an MCMV promoter regulated the expression of TSP-1 inserted in the EcoR I and Not I sites, and a CMV promoter regulated the expression of GFP gene, as a measure of transfection efficiency (Figure 1A). TCID50 was used to test the expression of Adv-TSP-1 revealing that the titers of the TSP-1 adenovirus (Adv-TSP-1) were 1×10^10^ PFU/ml.

The pHBAd-U6-GFP interference vector also had double-promoter to assess the transfection efficacy and expression of shRNA of TSP-1 expression. The double promoter plasmids contained a U6 promoter regulated the expression of shRNA of TSP-1 inserted in the BamH I and EcoR I sites, and a PCMV promoter regulated the expression of GFP gene, as a measure of transfection efficiency (Figure 1B). TCID50 was used to test the expression of Adv-TSP-1-shRNA revealing that the titers of the TSP-1-shRNA adenovirus Adv-TSP-1-shRNA were 2×10^10^ PFU/ml.

The BMSCs infected with adenovirus vectors were multiplicity of infection (MOI) of 50. Forty-eight hours after infection, the BMSCs were harvested and green fluorescence in the cytoplasm of BMSCs was identified by fluorescence microscope (Olympus Corp., Tokyo, Japan) (Figure 1C-1F). There were more than 80% of BMSCs showed green fluorescence, indicating high transduction efficiency of Adv-TSP-1 to BMSCs in the present study. We subsequently examined the levels of transferred gene expression of TSP-1 *in vitro* by western blot analysis and found the expression of TSP-1 was significantly increased in BMSCs+TSP-1 group, while almost no detectable level of TSP-1 in the BMSCs, BMSCs+MOCK and BMSCs-TSP-1 group (Figure 1G).

### Effects of TSP-1 on neurite outgrowth in VSC4.1 motor neurons after OGD injury

**Figure 2:**
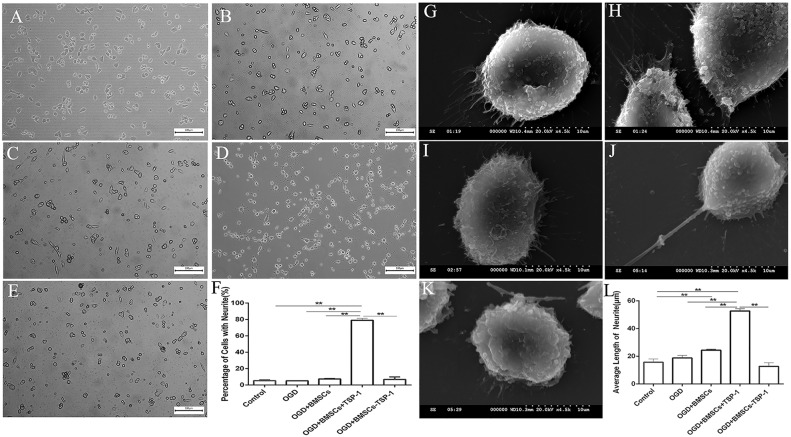
Effects of TSP-1-gene modified BMSCs on the neurite outgrowth in post-OGD VSC4 1 motor neurons. **(A-F)** Comparison of the percentage of VSC4.1 with neurite after OGD injury through the optical microscope, with (A) Control, (B) OGD, (C) OGD+BMSCs, (D) OGD+BMSCs+TSP-1 and (E) OGD+BMSCs-TSP-1, Scale bars: 100μm. (F) The percentage of the number of the cells with neurite in the Control, OGD, OGD+BMSCs, OGD+BMSCs+TSP-1 and OGD+BMSCs-TSP-1 group. ^**^P<0.01. **(G-L)** Comparison of the average length of neuritis after OGD injury through scanning electron microscopy, with (G) Control, (H) OGD, (I) OGD+BMSCs, (J) OGD+BMSCs+TSP-1 and (K) OGD+BMSCs-TSP-1, Scale bars:10μm. (L) The average length of cells with neurite in the Control, OGD+DMEM, OGD+BMSCs, OGD+BMSCs+TSP-1 and OGD+BMSCs-TSP-1 group. ^**^P<0.01.

For the purpose of detecting the influence of TSP-1 on neuronal neurite growth, the percentage of VSC4.1 with neurite and the length of neurite after OGD injury were measured by the optical microscope and scanning electron microscopy after co-cultured with different BMSCs, respectively. The results of optical microscope showed that the percentage of cells with neurite in OGD+BMSC+TSP-1 group was significantly more than that in the control group, OGD group, OGD+BMSCs group, and OGD+BMSCs-TSP-1 group (Figure 2A-2F, *P*<0.01). While there were no significant differences in the OGD group, OGD+BMSCs group and OGD+BMSCs-TSP-1 group (*P*>0.05). Then, we investigated whether the relative length of neurite would be specifically enhanced by TSP-1. The results of scanning electron microscopy demonstrated that the average length of neurite in the OGD VSC4.1 co-cultured with BMSCs+TSP-1 was significantly longer than that in the control group, OGD group, OGD+BMSCs group and OGD+BMSCs-TSP-1 group (Figure 2G-2L, *P*<0.01).

**Figure 3:**
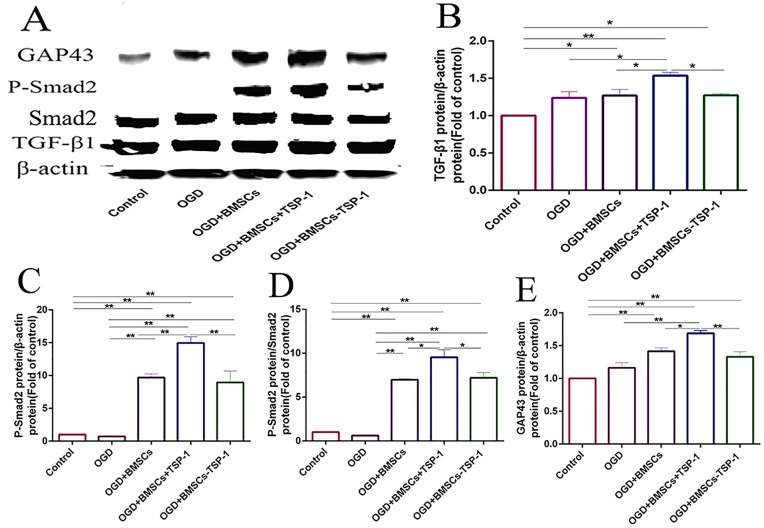
The expression of GAP-43, TGF-β1, p-Smad2 in VSC4 1 by western blot assay in the Control, OGD, OGD+BMSCs, OGD+BMSCs+TSP-1 and OGD+BMSCs-TSP-1 group. *P<0.05, ^**^P<0.01.

The growth-associated protein-43 (GAP-43) was a crucial protein which was associated with neurite growth. After co-culture of post-OGD VSC4.1 motor neurons with DMEM, BMSCs, BMSCs+TSP-1 and BMSCs-TSP-1, the expression of GAP-43 was measured by western blot assay. The results showed that the expression of GAP-43 in the OGD+BMSCs+TSP-1 group was significantly higher than that in four groups (Figure 3A, 3B, *P*<0.05). Then, we turned our attention to understanding whether TGF-β1/ p-Smad2 signaling pathway was involved in TSP-1 on promoting the expression of GAP-43 and neurite growth. The expression of TGF-β1 and p-Smad2 in the OGD injured VSC4.1 co-cultured with BMSCs+TSP-1 exhibited substantially the highest expression level of TGF-β1 (Figure 3A, 3C, *P*<0.01) and p-Smad2 ( Figure 3A, 3D, *P*<0.01) in all groups. However, the BMSCs-TSP-1 exhibited lower expression level of TGF-β1 and p-Smad2 than that in the OGD+BMSCs+TSP-1 groups (Figure 3, *P*<0.01).

### The BMSCs+TSP-1 treatment improved functional recovery in SCI rats

**Figure 4:**
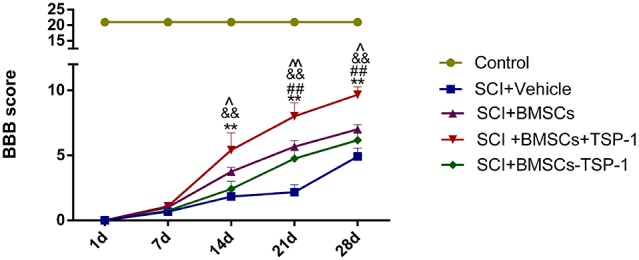
The Basso Beattie Bresnahan (BBB) scores at days 1, 7, 14, 21 and 28 after stem cells transplantation in all groups BBB score was significantly improved in SCI+BMSCs+TSP-1 group from 2 weeks to 4 weeks after transplantation. ^**^P<0.01, *versus* the SCI+vehicle group, ^##^*P*<0.01 *versus* the SCI+BMSCs group, ^&&^*P*<0.01 *versus* the SCI+BMSCs-TSP-1 group, ^*P*<0.05, ^^*P*<0.01, the SCI+BMSCs group versus the SCI+vehicle group.

The behavioral analysis was performed every week by using BBB locomotor scale. The BBB scores were displayed in Figure 4. After injury, the BBB scores of four groups decreased to 0 indicated successfully established spinal cord injury model at 1 day. The difference of BBB scores was no significance between each experimental group at 7 days (P>0.05). At 14 days after transplantation, the BBB scores were obviously improved in the SCI+BMSC+TSP-1 when compared to the SCI+vehicle group and SCI+BMSC-TSP-1 group (Figure 4, *P*<0.01). Furthermore, the BBB scores obviously elevated in the BMSC+TSP-1 treatment group compared to the SCI+vehicle, SCI+BMSC-TSP-1 and SCI+BMSCs group at 21 days, and 28 days after transplantation (Figure 4, *P*<0.01).

### The BMSCs+TSP-1 treatment counteracted apoptosis and promote cell survival in SCI rats

**Figure 5:**
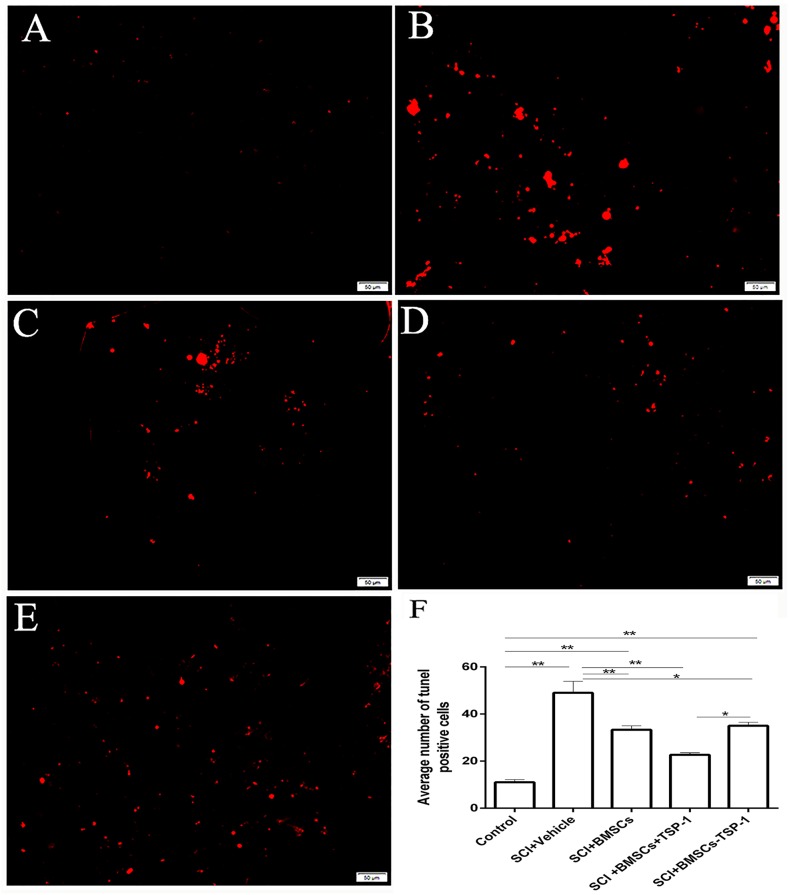
The number of apoptosis cells through TUNEL staining in injured spinal cord 3d after BMSCs transplantation Scale bars: 50μm. **(A)** Control group. **(B)** SCI + vehicle group. **(C)** SCI + BMSCs group. **(D)** SCI + BMSCs + TSP-1 group. **(E)** SCI + BMSCs- TSP-1 group. **(F)** The number of apoptosis cells in the control, SCI+vehicle, SCI+BMSCs, SCI+BMSCs+TSP-1 and SCI+BMSCs-TSP-1 group. *P<0.05, ^**^P<0.01.

We further tested whether the BMSCs+TSP-1 promoted cells survival through suppress apoptosis after stem cells transplantation. The terminal deoxynucleotidyl transferase dUTP nick end labeling (TUNEL) assay for apoptotic cell profiles was performed at 3 days after transplantation (Figure 5). The SCI+BMSCs+TSP-1 group rats showed fewer apoptotic cells than SCI+vehicle group and SCI+BMSCs-TSP-1 group after transplantation (Figure 5, *P*< 0.01). The number of apoptotic cells was significantly more in the SCI+vehicle and SCI+BMSCs-TSP-1 group than that in the control group (*P*<0.01). However, the SCI+BMSCs+TSP-1, SCI+BMSCs and SCI+BMSCs-TSP-1group showed less apoptotic cells in the spinal cord than that of the SCI group (Figure 5, *P*<0.01).

### The BMSCs+TSP-1 treatment increased the expression of TSP-1 and GAP-43 in SCI rats

**Figure 6:**
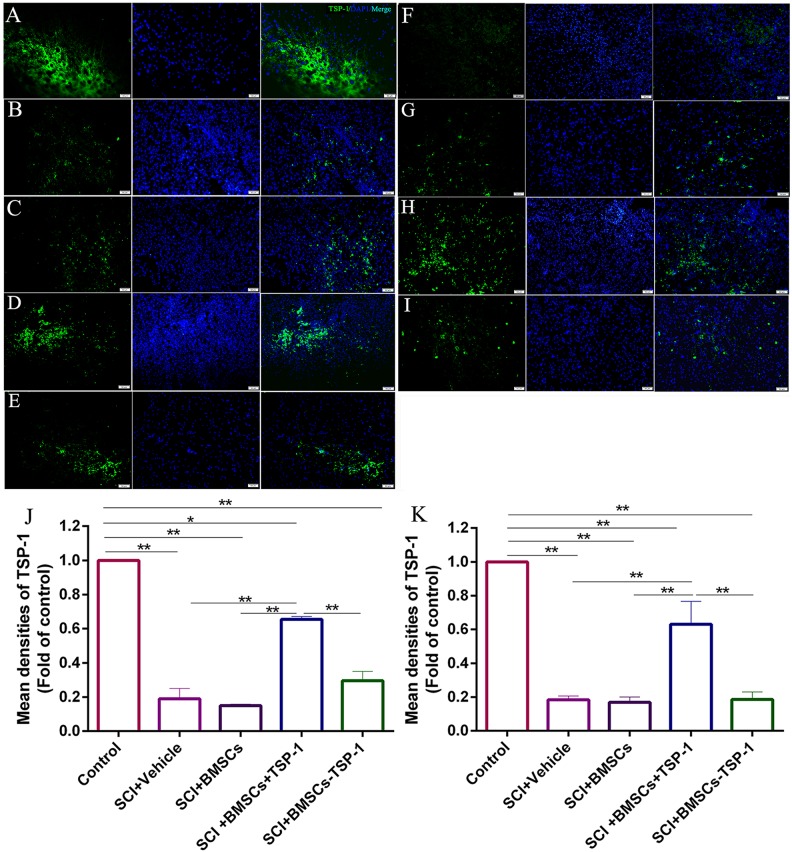
**(A-E, J)** The immunofluorescence of TSP-1 in injured spinal cord at 7d after BMSCs transplantation. (A) Control group. (B) SCI+vehicle group. (C) SCI+BMSCs group. (D) SCI+ BMSCs+TSP-1 group. (E) SCI+BMSCs-TSP-1 group. (J) The mean density of TSP-1 in SCI+vehicle, SCI+BMSCs, SCI+BMSCs+TSP-1 and SCI+BMSCs-TSP-1 group. *P<0.05, ^**^P<0.01, one-way ANOVA. Scale bars: 50μm. (A, F-I, K) The immunofluorescence of TSP-1 in injured spinal cord 28 d after BMSCs transplantation. (A) Control group. **(F)** SCI + vehicle group. **(G)** SCI + BMSCs group. **(H)** SCI+BMSCs+TSP-1 group. **(I)** SCI+BMSCs-TSP-1 group. **(K)** The mean density of TSP-1 in SCI+vehicle, SCI+BMSCs, SCI+BMSCs+TSP-1 and SCI+BMSCs-TSP-1 group. ^**^P<0.01.

To confirm the expression of TSP-1 in the spinal cord after cells transplantation, the immunofluorescence of TSP-1 in the anterior horn of the spinal cord was performed in each group at 7 days and 28 days after transplantation (Figure 6, *P*<0.01). The expression of TSP-1 was significantly higher in anterior horn of the rats in the SCI+BMSCs+TSP-1 group than that in the SCI+vehicle group, SCI+BMSCs group and the SCI+BMSC-TSP-1 group after 7 days transplantation (Figure 6A-6E, 6J, *P*<0.05). Meanwhile, the expression of TSP-1 in the SCI+BMSCs+TSP-1 was higher than that in the SCI+vehicle group, SCI+BMSCs group and SCI+BMSC-TSP-1 group after 28 days transplantation (Figure 6A, 6F-6I, 6K).

**Figure 7:**
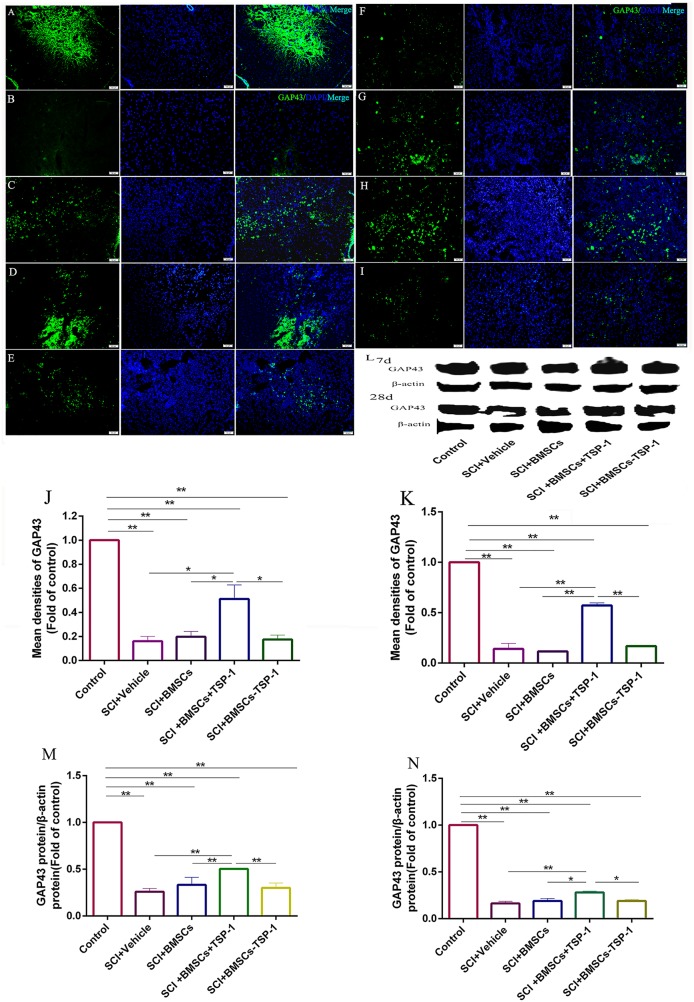
**(A-E, J, L, M)** The immunofluorescence and western blot assay of GAP-43 in injured spinal cord at 7d after BMSCs transplantation. Scale bars: 50μm. (A) Control group, (B) SCI + vehicle group, (C) SCI+BMSCs group, (D) SCI+BMSCs+TSP-1 group, (E) SCI+BMSCs-TSP-1 group, (J) The mean density of GAP-43 in SCI+vehicle, SCI+BMSCs, SCI+BMSCs+TSP-1 and SCI+BMSCs-TSP-1 group at 7d. ^**^P<0.01. (L) The western blot assay of GAP-43 in injured spinal cord at 7d and 28d after BMSCs transplantation. (M) The expression level of GAP-43 in SCI+vehicle, SCI+BMSCs, SCI+BMSCs+TSP-1 and SCI+BMSCs-TSP-1 group at 7d. (A, F-I, K, N) The immunofluorescence and western blot assay of GAP-43 in injured spinal cord at 28d after BMSCs transplantation. Scale bars: 50μm. (A) Control group, **(F)** SCI+vehicle group, **(G)** SCI+BMSCs group, **(H)** SCI+BMSCs+TSP-1 group, **(I)** SCI+BMSCs-TSP-1 group, **(K)** The mean density of GAP-43 in the SCI+vehicle, SCI+BMSCs, SCI+BMSCs+TSP-1 and SCI+BMSCs-TSP-1 group. ^**^P<0.01, *P<0.05. (N) The expression level of GAP-43 in SCI+vehicle, SCI+BMSCs, SCI+BMSCs+TSP-1 and SCI+BMSCs-TSP-1 group at 28d.

To evaluate the mechanism of the TSP-1 on functional recovery and neurite outgrowth in SCI rats, the expression of GAP-43, a crucial marker of neurite growth, was measured by immunofluorescence and western blot (Figure 7). The western blot assay revealed that the expression of GAP-43 was significantly decreased in the SCI+vehicle, SCI+BMSCs, SCI+BMSCs-TSP-1 and SCI+BMSCs+TSP-1 group compared with the control group after 7 days and 28 days transplantation. However, the SCI+BMSCs+TSP-1 treatment could significantly restore the expression of GAP-43 when compared with the SCI+vehicle, SCI+BMSCs and SCI+BMSCs-TSP-1 group after 7 days (Figure 7L, 7M, *P*<0.01) and 28 days (Figure 7L, 7N, *P*<0.01). The results of immunofluorescence of GAP-43 demonstrated the same trend towards that of western blot assay (Figure 7A-7K, *P*<0.05).

### The BMSCs+TSP-1 treatment activated TGF-β1/ Smad2 signal pathway in SCI rats

**Figure 8:**
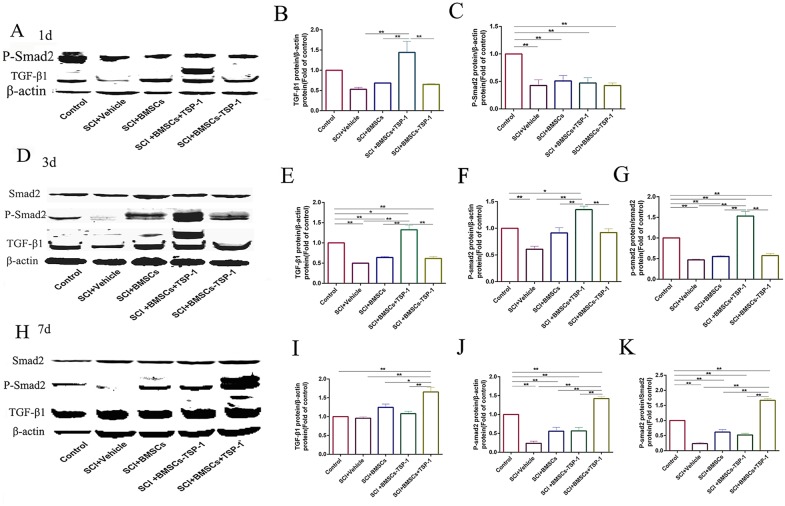
**(A, B, C)** The western blot assay of TGF-β1 and p-Smad2 in injured spinal cord at 1d after BMSCs transplantation. **(D, E, F, G)** The western blot assay of TGF-β1 and p-Smad2 in injured spinal cord at 3 days after BMSCs transplantation. **(H, I, J, K)** The western blot assay of TGF-β1 and p-Smad2 in injured spinal cord at 7days after BMSCs transplantation. ^**^P<0.01, *P<0.05.

The western blot analysis was conducted to assess the expressions of TGF-β1, p-Smad2 and Smad2 protein in the spinal cord of all groups. The results showed that the expression of TGF-β1 was significantly higher in the SCI+BMSCs+TSP-1 group than that of the SCI+vehicle group, SCI+BMSCs group and SCI+BMSCs-TSP-1 group after 1 day (Figure 8A, 8B, *P*<0.01), 3 days (Figure 8D, 8E, *P*<0.01), and 7 days transplantation (Figure 8H, 8I, *P*<0.05,).

There was no significant variation of the expression of p-Smad2 protein in all groups at one day after transplantation (Figure 8A, 8C, *P*>0.05,). However, the expression of p-Smad2 protein was significantly increased in the SCI+BMSCS+TSP-1 group compared with the SCI+vehicle group, SCI+BMSCS group and SCI+BMSCS-TSP-1 group at 3 days (Figure 8D, 8F, *P*<0.01), and 7 days after transplantation (Figure 8H, 8J, *P*<0.01). We also detected the ratio of phosphorylation of Smad2 variated in all groups. The results demonstrated that the ratio of phosphorylation of Smad2 was significantly increased in the SCI+BMSCS+TSP-1 group when compared with the SCI+vehicle group, SCI+BMSCS group and SCI+BMSCS-TSP-1 group after 3 days (Figure 8D, 8G, *P*<0.01), and 7 days transplantation (Figure 8H, 8K, *P*<0.01).

## DISCUSSION

The SCI leads to neurological complications and eventually paraplegia or quadriplegia. The presence of extracellular inhibitory factors and the lack of neurotrophic factors are major contributors to few neurite regeneration after spinal cord injury [7, 28, 29]. Interestingly, the present results demonstrated that TSP-1 overexpression of BMSCs could promote neurite outgrowth and functional recovery after spinal cord injury. Our results for the first time suggested that the BMSCs+TSP-1 implanted at a T10 injured site robustly restore locomotor function and promote neurite outgrowth by activating TGF-β1/p-Samd2 pathway. Notably, neurite outgrowth was the frequently pursued CNS regenerative goal in treating SCI. Our results collectively introduced a different experimental platform of TSP-1 modified stem cell-based therapy for the injured spinal cord.

We and other scientists already found that transplantation of BMSCs could significantly improve the hind limb locomotor function in experimental models of SCI by modulating diverse physiopathological events, such as inflammatory reaction, oxidative stress, apoptosis, angiogenesis [20, 27]. However, the researchers find that BMSCs do not persist and integrate following transplantation; overall, functional recovery post treatment remains limited to modest improvements [30, 31]. Prior studies have suggested that the adult spinal cord does not regenerate, predominantly due to the inability of neurons to regenerate neurite for the inhibitory milieu of the glial scar of injured spinal cord lesion [32–34]. How to help BMSCs to promote neurite regeneration became the key therapeutic mechanisms after SCI.

The TSPs were originally discovered in human blood platelets, released from astrocytes and promoted neurite formation and adhesion in neurons [8, 10]. Recently, the studies revealed that TSP-1 was an important secretory astrocytic protein that could promote synapse formation and neurite outgrowth [9, 10, 35]. Here, we transferred TSP-1 to BMSCs then cultured with OGD injured VSC4.1 motor neurons. We found that the BMSCs+TSP-1 could prolong the length of neurite and increase GAP-43 expression. Silencing the expression of TSP-1 led to the complete loss of the neurite outgrowth-stimulating function. TSP-1 expressed in astrocytes in normal spinal cord guarantees the growth of neurite. Neurons and astrocytes are injured after SCI, which leads to insufficient amounts of TSP-1 to promote the outgrowth of neurite. After TSP-1 overexpression BMSCS were transplanted to the injury site, the expression level of TSP-1 was increased, then to improve the neurite outgrowth, even in the injured spinal cord. Furthermore, the BBB scores showed that SCI could lead to a decline in the BBB scores compared to that of the control group. The BMSCs treatment effectively increased the BBB scores in SCI rats. However, the BBB scores of the BMSCs+TSP-1 group were higher than that of only the BMSCs and SCI+BMSCs-TSP-1 group. Then we explored the possible mechanisms of the BMSCs+TSP-1 protective effects on SCI by analysis the downstream protein TGF-β1.

The TGF-β1 was a multifunctional cytokine with anti-inflammatory, reparative and neuroprotective functions. Previous studies have shown that the TGF-β1 signaling plays the critical roles in astrocytes and microglia under physiological and injury conditions [36]. In addition, the TGF-β1 signaling has been shown to regulate specification in the embryonic cortical neurons and increases neurogenesis in the adult dentate gyrus [37]. These studies support the important role of the TGF-β1 signaling in neurite outgrowth and maintenance [38, 39]. Several factors can activate latent TGF-β1, including proteases, integrins, latent TGF-β1-binding protein, oxidant modification of the latent complex, and the matricellular protein TSP-1. The TSP-1 binds to the latent TGF-β1 complex through defined sequences and induces a conformational change in the latent TGF-β1 complex, rendering it biologically active [40–42]. The present results demonstrated that the TGF-β1, activated by the TSP-1, and increased the phosphorylation of smad2 in the BMSC+TSP-1 treated OGD injured VSC4.1 motor neurons and injured spinal cord of SCI rats. Studies on neurite outgrowth and other changes in neuronal morphology have provided an indirect evidence for the interrelationship between the TSP-1/TGF-β1 and injured neurons. This evidence strongly suggests that the TSP-1 may participate in neurite outgrowth, in part, by regulation TGF-β1/p-Smad2.

In conclusions, our results provided an important mechanistic understanding on BMSCs+TSP-1 participating in neurite outgrowth by activating TGF-β1/p-Smad2 to improve the functional recovery. Further studies will continue to explore the TSP-1 modified BMSCs might be used therapeutically in patients with SCI.

## MATERIALS AND METHODS

### Experimental animals

A total of ninety Sprague-Dawley (SD) rats (220–250g) and ten 3-week-old SD rats were purchased from Zhejiang experimental animal center (Hangzhou, China). All rats were fed in the SPF laboratory separately at a room temperature of 25 ± 3°C, a relative humidity of 55%~75%, and free access to food and water. BMSCs were extracted from ten 3-week-old SD rats. All experimental procedures were approved by the Institutional Animal Care and Use Committee of Zhejiang University. All efforts were made to minimize the number of animals used and their suffering.

### Primary BMSCs cultured, production of adenovirus and infection of BMSCs

The BMSCs were prepared from the femurs of ten 3-week-old SD rats as our previously reported [20, 21]. The rat TSP-1 gene (Ref sequence: NM_001013062) was synthesized by the Han Bio Co. LTD (Shanghai, China) and confirmed by sequencing. The expression vector pHBAdv-MCMV-GFP had double-promoter to assess the transfection efficacy and expression of TSP-1. The double promoter plasmids contained a MCMV promoter regulated the expression of TSP-1 inserted in the EcoR I and Not I sites, and a CMV promoter regulated the expression of GFP gene to as a measure of transfection efficiency. LipofiterTM (Han Bio Co. LTD Shanghai, China) was used to create the adenovirus of pHBAdv-MCMV-GFP-TSP-1 in HEK293 cells. TCID50 was used to test the titers of the adenovirus.

The shRNA of rat TSP-1 gene was designed and synthesized by the Han Bio Co.LTD (Shanghai, China). The pHBAd-U6-GFP interference vector also had double-promoter to assess the transfection efficacy and expression of shRNA of rat TSP-1 gene expression. The double promoter plasmids contained a U6 promoter regulated the expression of shRNA of TSP-1 inserted in the BamH I and EcoR I sites, and a PCMV promoter regulated the expression of GFP gene to as a measure of transfection efficiency. LipofiterTM was used to create the adenovirus of pHBAdv-U6-GFP-shRNA of TSP-1 in HEK293 cells. TCID50 was used to test the titers of the adenovirus.

The BMSCs were infected with adenovirus vectors, and the multiplicity of infection (MOI) was 50. Forty-eight hours after inoculation, the infected TSP-1 of BMSCs were subjected to a fluorescence microscope (Olympus Corp., Tokyo, Japan) and the Western blot analysis to detect the TSP-1 expression. TSP-1 overexpression BMSCs were named as BMSCs+TSP-1 and BMSCs with shRNA of TSP-1 were named as BMSCs-TSP-1.

### Oxygen–glucose deprivation (OGD) model and co-culture of post-OGD VSC4.1 motor neurons with TSP-1 gene-modified BMSCs

The ventral spinal cord 4.1 (VSC4.1) motor neuron cells were cultured in RPMI 1640 medium with 10% (V/V) fetal bovine serum (FBS, Gibco, Invitrogen) and 1% penicillin and streptomycin (Gibco, Invitrogen) at 37 °C with 5% CO_2_ in a fully humidified incubator. Briefly, VSC4.1 motor neurons were cultured in glucose-free Hanks’ balanced salt solution in a sealed hypoxic GENbag fitted with a catalyst (BioMèrieux, Marcy I’Etoile, France) to scavenge free oxygen, then subjected to 0% oxygen condition for 4 hours. As the Non-OGD control, Cells cultured in Hanks’ balanced salt solution containing the normal concentration of glucose with 5% CO_2_.

BMSCs (5 × 10^5^/well) were cultured in the insert chamber of the 6-well 0.4μm trans-well system and co-cultured with 5 × 10^5^/well VSC4.1 motor neurons. The insert chambers were removed after 48 hours and VSC4.1 motor neurons were exposed to 4 hours OGD, and 20 hours re-oxygenation. Then VSC4.1 motor neurons were collected for western blot assay and scanning electron microscopy observation.

### Scanning electron microscopy

Scanning electron microscopy was carried out to observe neurite growth in motor neurons. After post-OGD VSC4.1 motor neurons co-cultured with the BMSCs, BMSCs+TSP-1 and BMSCs-TSP-1, the slides climbed fully of VSC4.1 motor neurons were taken out. After fixed with 5% glutaraldehyde, a series of dehydration with an incremental concentration of acetone at 25 °C as follows: 25% acetone 15 min; 50% acetone 15 min; 75% acetone 15 min; 95% acetone 15 min; 100% acetone 15 min, the neurons were mounted on copper grids coated with formvar, and stained with lead citrate. Images were obtained using a scanning electron microscope (HITACHI S-3000N, Japan). The length of neurite in VSC4.1 after OGD injury were measured by scanning electron microscopy after co-cultured in all groups. The average length of neurites in each group was obtained by Image-Pro Plus 6.0 to measure the length of neurites in four electron microscope photographs, then the data was averaged.

### Spinal cord injury model and cell transplantation

The rats were divided into five groups: the control group, SCI+vehicle group, SCI+BMSCs group, SCI+BMSCs+TSP-1 group and SCI+BMSCs-TSP-1 group (n = 18/group). The rats were anesthetized with sodium pentobarbital (40 mg/kg, i.p.), and a weight of 10g was dropped from a height of 5cm on the exposed spinal cord to produce a moderate contusion on T10 level. The rats of the control group were exposed spinal cord but no injury. After injury, the 10μl cell culture medium DMEM including a million BMSCs+TSP-1, BMSCs-TSP-1 or BMSCs were injected into the epicenter of the injured spinal cord using an electrode microneedle as the SCI+BMSCs group, SCI+BMSCs+TSP-1 group and SCI+BMSCs-TSP-1 group respectively. While the same quantity of cell culture medium DMEM was injected into the SCI rats as the SCI+vehicle group after contusion.

Following the transplantation, all rats received daily subcutaneous ampicillin (100 mg/kg) for the first 7 days to prevent infection. Before reflexive urination returned, the bladders were manually voided twice daily.

### Assessments of motor function

The motor function was assessed by Basso, Beattie, and Bresnahan (BBB) locomotor scale, a measure of hindlimb function ranging scale from 0 (complete paralysis) to 21 (normal locomotion). Briefly, all rats were observed and assessed the relevant indicators of hind limb motor function and physical control function in an open field for 2-3 min at 1, 7, 14, 21 and 28 days after SCI.

### Tissue processing

The rats were anesthetized at 1d, 3d, 7d and 28d after BMSCs transplantation respectively, for the protein analysis, the injured spinal cord segment (1 cm length including before and after the injury site 0.5 mm) was taken out. The rest rats were perfused with normal saline, and 4% paraformaldehyde and the injured spinal cord segment was taken out for frozen section preparation. The specimen was transferred to a solution containing 30 % sucrose in 0.1 M PBS (pH 7.4) at 4 °C, until it stayed at the bottom of the container, and the spinal cord segment containing the entire injury site for longitudinal sections were embedded in OCT compound (Sakura Finetechnical, Tokyo, Japan) and frozen. All specimens were cut into the 20 um thickness sections by Cryostat Microtome (Leica, Germany) and the sections were mounted on gelatin-coated slides.

### TUNEL staining assay

The apoptotic cells were analyzed by an in situ One Step TUNEL Apoptosis Assay Kit (Beyotime Institute of Biotechnology, Nantong, Jiangsu, China), following the procedure specified by the manufacturer. DNA fragmentation of nuclei in the injured areas was stained by in situ TdT-mediated dUTP nick end labeling (TUNEL) method. Apoptotic cells showed red fluorescence. Every eight tissue sections were used for one animal and photographed at a ×200 picture.

### Immunofluorescence staining

The immunofluorescence analysis was used to identify the expression of TSP-1 and GAP-43 in all groups. The sections were incubated overnight with the primary antibody at 4 °C, washed three times with PBS, and then incubated with the fluorescent-conjugated secondary antibody. Slides were incubated with DAPI for 5 min, cover slipped, and all pictures were captured at the same area of tissue sections using a fluorescence microscope (Olympus BX61). Area measurements were performed using the Image-Pro Plus 5.0 image analysis software (Media Cybernetics Inc., Atlanta, GA, USA).

### Western blot

The primary antibodies were used as follows: TSP-1 antibody (1:1000, Cell signaling technology, USA), GAP-43 antibody (1:1000, Cell signaling technology, USA), TGF-β1 antibody (1:1000, Cell signaling technology, USA), smad2 antibody (1:1000, Cell signaling technology, USA), p-Smad2 antibody (1:1000, Cell signaling technology, USA), and β-actin antibody (1:5000; Sigma, USA). After several washes with TBS-T, blots were incubated with infrared labeled secondary antibody (Li-COR Biosciences). Immunoblot bands were visualized and analyzed by using Odyssey CLx infrared imaging system (LI-COR^®^ Biosciences, USA).

### Statistical analysis

All the data were presented as mean ± SEM. Statistical analysis was performed by using one-way analysis of variance (ANOVA) or two-way analysis of variance (ANOVA). Differences were considered significant at P < 0.05.
